# A novel free-electron laser single-pulse Wollaston polarimeter for magneto-dynamical studies

**DOI:** 10.1063/4.0000104

**Published:** 2021-06-18

**Authors:** Antonio Caretta, Simone Laterza, Valentina Bonanni, Rudi Sergo, Carlo Dri, Giuseppe Cautero, Fabio Galassi, Matteo Zamolo, Alberto Simoncig, Marco Zangrando, Alessandro Gessini, Simone Dal Zilio, Roberto Flammini, Paolo Moras, Alexander Demidovich, Miltcho Danailov, Fulvio Parmigiani, Marco Malvestuto

**Affiliations:** 1 Elettra-Sincrotrone Trieste S.C.p.A., Strada Statale 14 - km 163.5 in AREA Science Park, Basovizza, 34149 Trieste, Italy; 2 Università degli Studi di Trieste, Via A. Valerio 2, 34127 Trieste, Italy; 3 Istituto Officina Dei Materiali-CNR, Strada Statale 14 - km 163.5 in AREA Science Park, Basovizza, 34149 Trieste, Italy; 4 CNR-ISM, Istituto di Struttura della Materia, Via del Fosso del Cavaliere 100, I-00133 Roma, Italy; 5 CNR-ISM, Istituto di Struttura della Materia, Strada Statale 14 - km 163.5 in AREA Science Park, Basovizza, 34149 Trieste, Italy; 6 International Faculty, University of Cologne, 50937 Cologne, Germany

## Abstract

Here, we report on the conceptual design, the hardware realization, and the first experimental results of a novel and compact x-ray polarimeter capable of a single-pulse linear polarization angle detection in the extreme ultraviolet photon energy range. The polarimeter is tested by performing time resolved pump–probe experiments on a Ni_80_Fe_20_ Permalloy film at the M_2,3_ Ni edge at an externally seeded free-electron laser source. Comparison with similar experiments reported in the literature shows the advantages of our approach also in view of future experiments.

## I. INTRODUCTION

The availability of ultrashort electromagnetic pulses started the exploration of previously unaccessible phenomena in condensed matter physics. The generation of stable and intense sub-100 fs laser pulses was made possible by the discovery of chirped-pulse amplification^1^ and self-mode-locking^2^ in the early 1990s. Thanks to the high electric fields of the pulses, the frequency of the electromagnetic radiation could be converted via nonlinear effects from the visible to the mid-infrared (MIR) range. The field of femtosecond optical spectroscopy was developed also thanks to these aforementioned technical advancements.

While the femtosecond optical spectroscopy initially could not extend to the x-ray regime, immediate and huge were the effort spent by scientific community to fill this gap.^3^ The effort was finally capped off by the launch of the free electron laser (FEL) Linear Coherent Light Source (LCLS, Stanford, USA) in 2010, with pulses generated down to 1.2 Å.^4^ The development of the seeded-FEL Free Electron laser Radiation for Multidisciplinary Investigations (FERMI, Trieste, Italy) in 2012^5^ represented a further milestone in terms of polarization control,^6^ stability, and coherence.^7,8^ FERMI works in the extreme ultraviolet (EUV) range, from 100 down to 4 nm.^9^ Several important experimental techniques had being developed within the FERMI laboratories, such as EUV transient-grating (TG) and EUV magnetic-TG,^10,11^ simultaneous time, and two-color spectroscopy.^12,13^ Motivated by the rapidly growing field of the so-called femtomagnetism,^14^ we have designed, built, and commissioned a novel and effective polarimeter for ultrafast magneto-optical experiments in the EUV photon energy range.

The magnetization status of a compound can be accurately determined, with a femtosecond resolution, by several well-established techniques. As reviewed in Yamamoto and Matsuda,^15^ the most effective and frequently used time resolved techniques are (i) in the visible range, magneto-optic Kerr effect (MOKE) and Faraday effect (FE) experiments and (ii) in the soft x-ray regime, x-ray circular dichroism (XMCD). Recently, resonant MOKE (RMOKE) was demonstrated in the EUV range.^16^ RMOKE refers to a MOKE experiment performed at photon energies close to an element absorption edge. A detailed comparison of RMOKE and XMCD is given in Ref. 15, where the advantages of RMOKE are enlightened, namely, M-edge feasibility, measurement of polarization rotation and ellipticity, use of linear polarization, giant Kerr, element sensitivity, and sub-picosecond time-resolution. Moreover, we expect advantages similar to RMOKE for giant Faraday^17^ and giant natural birefringence.^18^ Important is also to underline that XMCD experiments at FEL facilities like FERMI, where circularly polarized photons can be generated with a very high degree of polarization, are well established. Here, we want to stress the potential of RMOKE as a complementary technique to XMCD.

Yamamoto and Matsuda^15^ show that the Kerr and the Faraday effects in the EUV range, corresponding to the M-edge of the 3d transition metals, are up to 100 times larger than in the visible range and have the advantage of being element sensitive. Additionally, compared to XMCD experiments performed at the L-edges, the reflectivity in the EUV is higher, and the use of linear polarization avoids complications associated with the use of circular polarization. In the first RMOKE experiment,^16^ the magnetization-induced x-ray polarization rotation in polar MOKE geometry was detected by the rotating-analyzer ellipsometer (RAE), an instrument specifically developed for the experiment. The RAE is composed of a multilayer mirror, placed at the Brewster angle, that collects the x-ray pulse reflected by the sample rotating along the incoming beam axis. Note that similar polarimeters had being already utilized in literature^19–21^ for similar purposes. The intensity of the x-ray pulse reflected by the multilayer mirror as a function of the rotation angle can be described as a phase-shifted squared sinus wave, where the phase-shift corresponds to the Kerr rotation angle. Although the authors showed excellent measurement on both Ni and Fe films at the respective M-edges, the measurement involves mechanical displacement of several elements and it is time consuming, since a full detector rotation acquisition is needed for a single polarization angle estimate.

We developed a polarimeter, resembling the Wollaston balanced-photodetection scheme in the visible range, capable of single-pulse linear polarization angle detection in the EUV energy range. The Wollaston polarimeter for x-ray FEL sources (TONIX) is currently installed at the MagneDyn beamline^22^ at the FERMI FEL in Trieste, Italy, and is available for users. In this report, first, we describe the conceptual design and the mechanical realization of the TONIX; second, we test the instrument by performing time-resolved magnetization dynamic studies on a Ni_80_Fe_20_ Permalloy.

## II. TONIX DESIGN AND REALIZATION

**FIG. 1.**
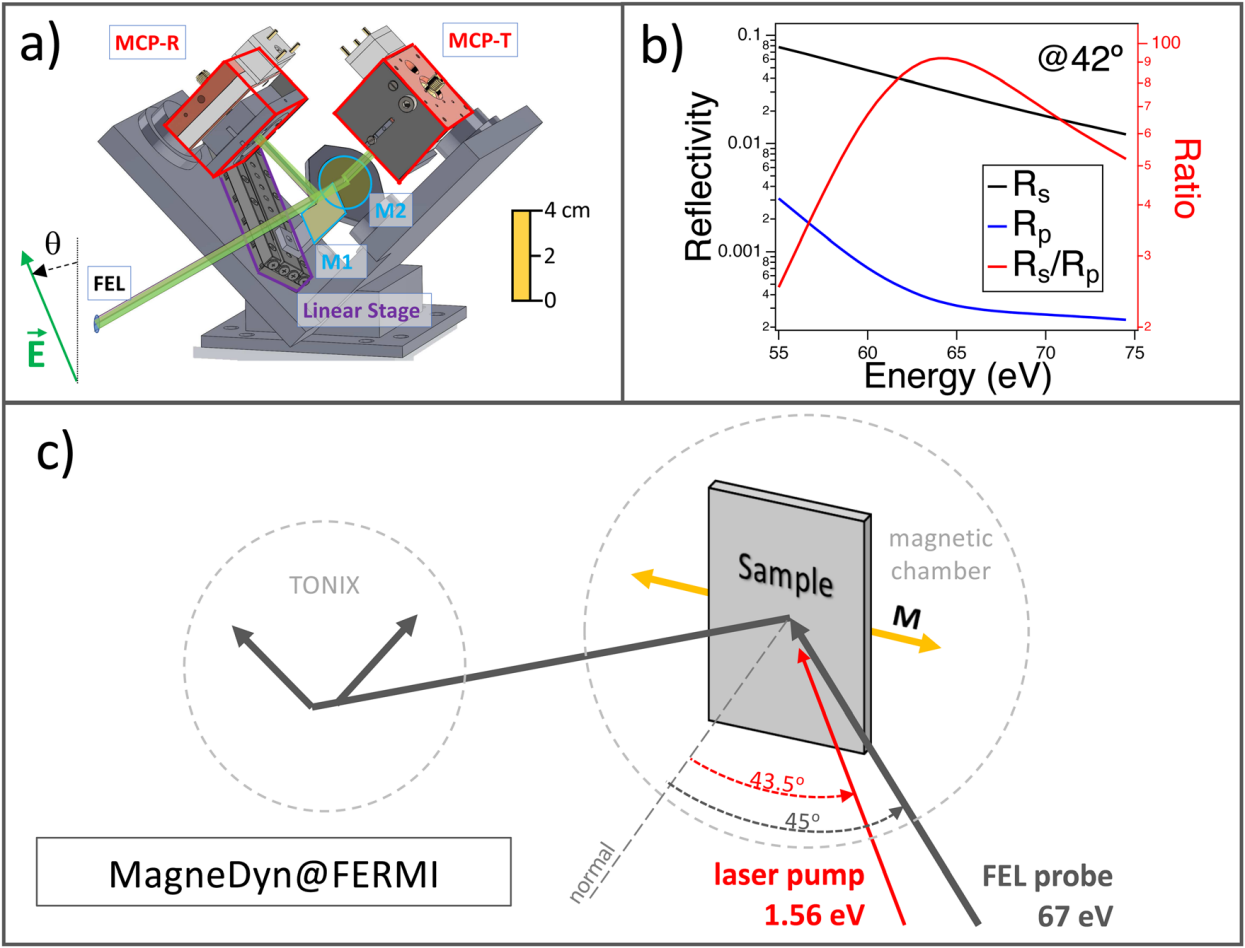
(a) TONIX 3D technical drawing, front side view. The schematics of the beam splitting and the MCP mounts are also displayed. A ruler is plotted on the right side to display the TONIX size. The green arrow represents the electric field vector $\vec{E}$ of the incoming x-ray pulse, and *θ* is the angular deviation from the vertical or horizontal axis. (b) Nb mirror *s*- and *p*-polarization reflectivity and extinction ratio at a fixed angle of incidence of 42° in the range 60–75 eV, corresponding to the M_2,3_ edge in Ni. (c) Scheme of the time-resolved experiment setup at the MagneDyn beamline at FERMI FEL (Trieste, Italy). The angles of incidence of the FEL probe and of the laser pump are, respectively, 45° and 43.5°. The pump laser photon energy is 1.56 eV (794 nm). The FEL and the laser spot sizes are approximately 100 *μ*m in sigma, but be reduced down to 15 *μ*m.^22^ The laser pump fluence ranges from 0.15 to 63 mJ/cm^2^.

In optics, polarization sensitive balanced photodetection is performed by (i) decomposition of the incoming beam into two components orthogonal in polarization and equal in intensity with the use of high-quality polarizers (e.g., the Wollaston polarizer); (ii) simultaneous detection of the intensity of the two components; (iii) polarization angle reconstruction by the difference of the two signals. In the optical regime, there are other polarization-sensitive techniques based, for instance, on active phase modulators (like the photoelastic modulator PEM^23^), but such devices are not available for the x-ray regime. In the TONIX, the decomposition and the polarization selection are performed by two mirrors placed at the Brewster angle, mounted on two orthogonal planes of incidence. A 3D drawing of the assembly is shown in Fig. 1(a). The first mirror M1, aside from reflecting the beam to the first multichannel plate (MCP)-reflection (MCP-R), splits the beam in half. The second mirror M2 reflects the transmitted beam to the second MCP-transmission (MCP-T). M1 is mounted on a linear translation stage in order to fine-tune the beam splitting in exact half. On the same motorized linear stage, an EUV fluorescence screen (yttrium aluminum garnet, YAG) is mounted for alignment purposes (not shown in the figure). Such geometry is chosen to maximize the sensitivity for incoming linear vertical or linear horizontal x-ray beam polarization states. In order to match the Brewster angle of the two mirrors in selected energy ranges, the mirrors M1 and M2 can be tuned from 20° to 60° in angle of incidence simply by tilting the mirror mounts on their optical base. Currently, the axis rotation of the mirrors is not motorized but fixed before the experiment, since the Brewster angle has weak dependence on photon energy variations of ±10 eV.

## III. MIRROR DEPOSITION

The mirrors are composed of 100 nm of Nb films deposited on Si substrates by MBE. Nb is a metal with good reflectivity in the EUV range (∼3%) and good extinction ratio of the *p*- with respect to the *s*-polarization ( $R_{s}/R_{p}∼$ 10^2^). In Fig. 1(b), we show the mirror reflectivities *R_s_*, *R_p_*, and the extinction ratio $R_{s}/R_{p}$, all calculated at the angle of incidence of 42°, which is the Brewster angle for ∼65 eV photons. In the future, for specific experiments performed at photon energies above 100 eV, we will adopt multilayer mirrors in order to enhance the reflectivity of the TONIX polarimeter mirrors.

## IV. MCP ASSEMBLY, POWER SUPPLY

The front plate of the MCPs is powered using HV Flex power supplies,^24^ feeding voltages ranging from −700 to −500 V. The back plate of the MCPs is fed with −20 to −10 V. The output electrons from the MCP are collected by an anode at 0 V, and the signal is amplified by a 22 dB amplifier.

## V. SIGNAL PROCESSING AND METHODS

**FIG. 2.**
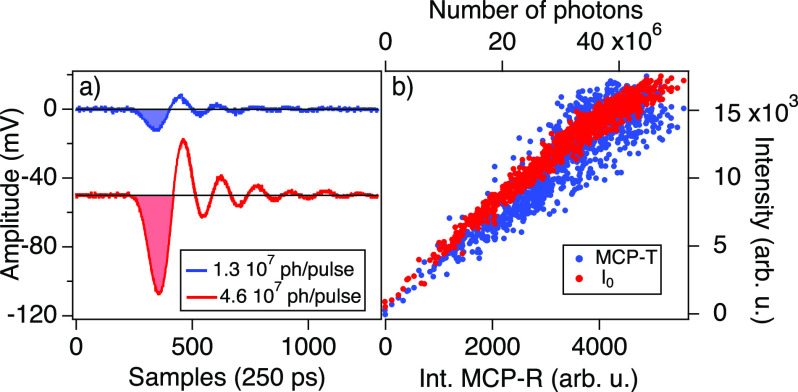
(a) Digitalized MCP signals of two FEL pulses at 67 eV. The label indicates the number of photons per pulse. The signal of each MCP is the integral of the colored region shown in the graph. (b) Pulse-to-pulse dependence of both the MCP-T signal and the I_0_ FEL intensity monitor as a function of the second MCP-R signal. The top axis reports the incoming number of photons of each FEL pulse.

The amplified signals from the two MCPs are digitalized using a CAEN v1761,^25^ at a sampling rate of 4 GHz. For each FEL pulse, the digitizer returns two digitalized traces. As an example, in Fig. 2(a), we plot the digitalized traces *f*(*t*) of two EUV pulses having different intensities, as function of the sampling time. The label of each curve corresponds to the number of photons of the incoming pulse. The ringing effect is due to the impedance mismatch. We obtain the pulse intensity as the integral of the negative first half wave oscillation after a constant background subtraction, as shown in Fig. 2(a) by the colored area under each curve. The pulse intensities can be normalized by *I*_0_, the photocurrent amplitude of a beamline mirror. In Fig. 2(b), we plot the pulse-to-pulse MCP-T signal as a function of MCP-R showing reasonable linearity. For comparison, we plot in the same graph the dependence of the *I*_0_ FEL intensity monitor on the MCP-R intensity.

Given a single FEL pulse, we obtain the MCP-R and MCP-T signals, respectively, *S_R_* and *S_T_*. The polarization angle *θ* and the reflectivity R are calculated by^26^

$$\begin{array}{c} \theta =\;\frac{1}{2}\;\frac{\left(S_{T}-S_{R}\right)}{\left(S_{T}+S_{R}\right)},\\ R\propto \;(S_{T}+S_{R}). \end{array}$$ (1)

The polarization angle *θ*, as shown in Fig. 1(a), represents the deviation in radians from the vertical or the horizontal axis of the electric field of the x-ray pulse.

## VI. SAMPLE PREPARATION

The 100 nm Ni_80_Fe_20_ Permalloy magnetic film is deposited on a Si substrate by pulsed laser deposition with the use of a Ni and a Fe target (99.98%). Oxidation is prevented with the use of a Pt capping layer of 5 nm.

## VII. STATIC AND TIME-RESOLVED KERR ROTATION

**FIG. 3.**
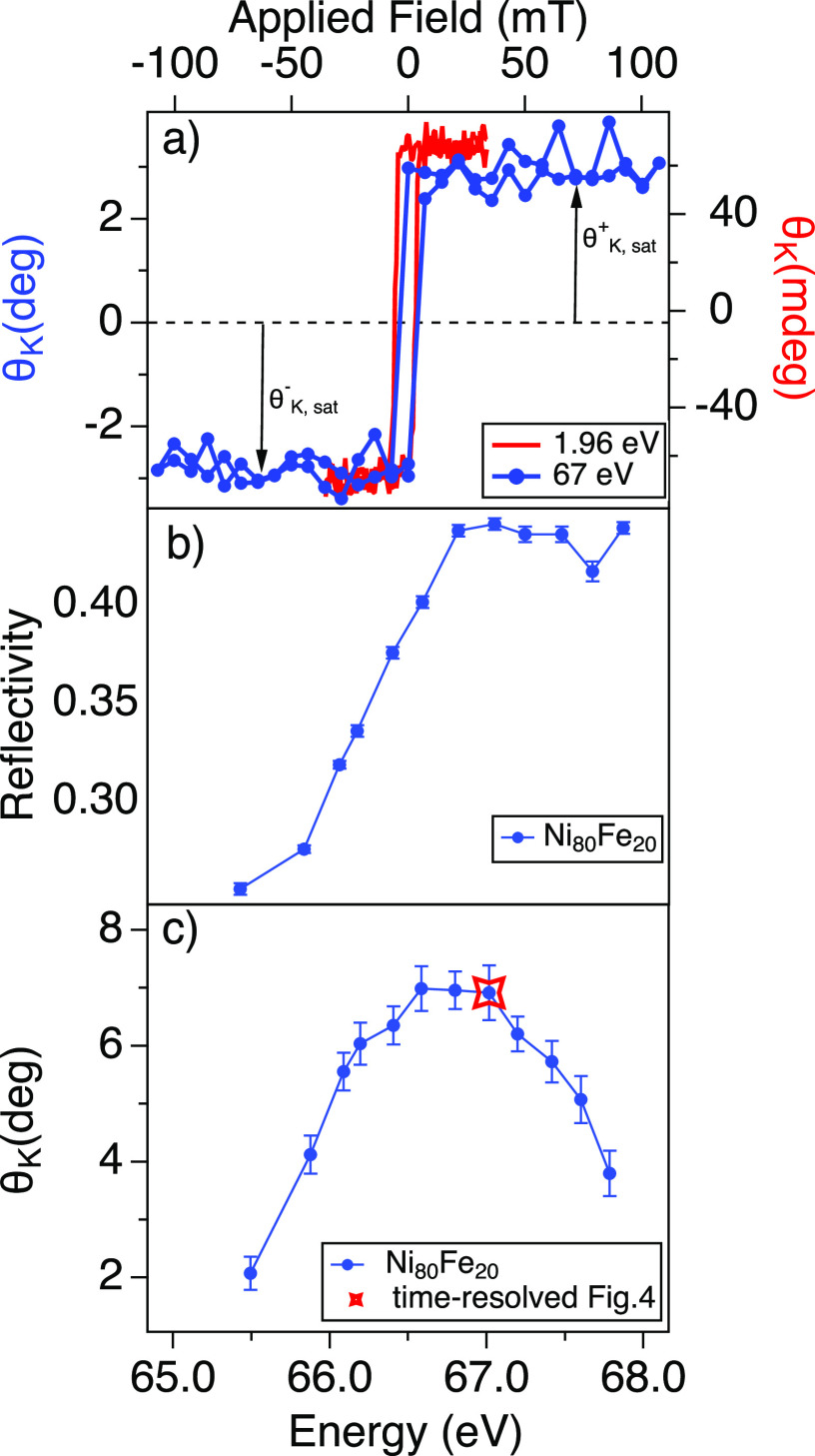
(a) Magnetization hysteresis on Ni_80_Fe_20_ at the M_2,3_ Ni edge (67 eV, blue) and in the visible range at 1.96 eV (632 nm, red). (b) Ni_80_Fe_20_ reflectivity corrected for reflectivity response of the Nb mirrors of the TONIX polarimeter and (c) Kerr rotation at saturation as function of the FEL photon energy; the starred data-point corresponds to the FEL energy of the time-resolved experiments shown in Fig. 4.

We tested the TONIX operation through RMOKE measurements on the Ni_80_Fe_20_ Permalloy film with the FERMI FEL pulses tuned at the Ni M_2,3_ edge (Ni 3p, 67 eV). The experimental scheme is illustrated in Fig. 1(c). First, we recorded a static Kerr hysteresis at 67 eV. By working at applied fields of the order of 50 mT, the Kerr angle measures the in-plane magnetic component laying on the optical plane (longitudinal MOKE). As it is shown in Fig. 3(a), the measurement is in agreement with the visible MOKE collected at 1.96 eV (632 nm) on the same sample and with the same experimental configuration. With the TONIX, the hysteresis curve is collected in approximately 5 min (approximately 15 000 pulses at 50 Hz). In Figs. 3(b) and 3(c), we show, respectively, the sample reflectivity and the Kerr rotation as a function of the FEL photon energy. Note that the sample reflectivity is corrected by the Nb mirrors response.

**FIG. 4.**
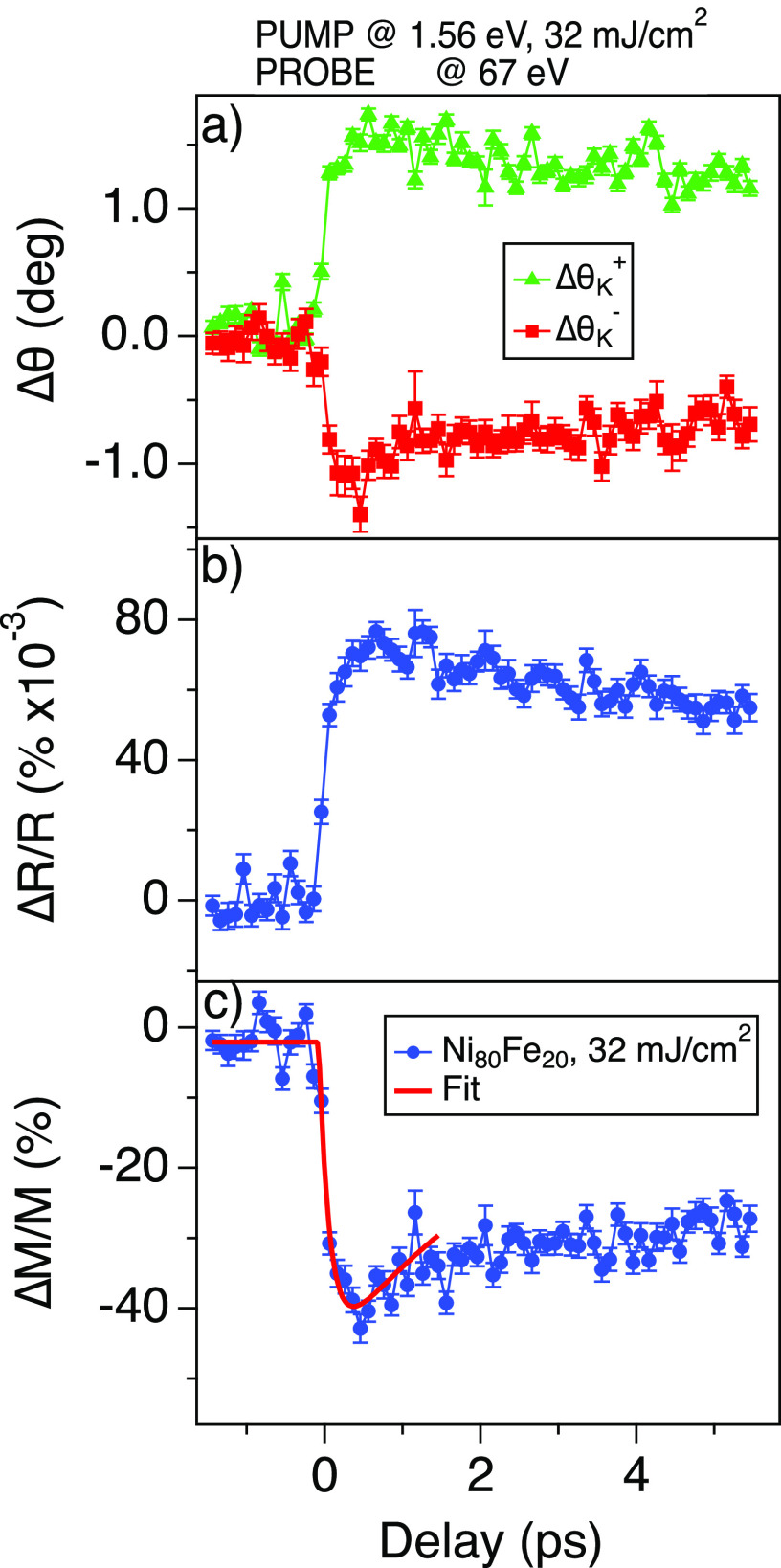
(a) Kerr rotation time-traces at positive, $\Delta \theta_{K}^{+}(t)$, and negative, $\Delta \theta_{K}^{-}(t)$, applied magnetic fields (±50 mT). (b) Reflectivity changes time-evolution. (c) Reconstructed magnetization dynamics on Ni_80_Fe_20_ (blue dots) and fit of the data with the function described in Eq. (3) (red line).

We also carried out time-resolved demagnetization experiments using the 1.56 eV (794 nm) laser pump excitation, probing the Kerr rotation at 67 eV. As shown in Fig. 1(c), the FEL and the laser pulses hit the sample at an angle of incidence of 45° and 43.5°, respectively. The circular Gaussian spots have dimensions of, respectively, 80 and 100 *μ*m in sigma. The FEL spot size can be adjusted, thanks to the Kirkpatrick–Baez mirrors, down to the resolution limit below 15 *μ*m at 62 eV.^22^ The pulse energy of the horizontally polarized FEL is kept below 1 *μ*J, and the laser pump pulses range between 0.1 and 40 *μ*J. Correspondingly, the FEL fluence is ∼2.5 mJ/cm^2^, while the pump laser fluence ranges from 0.15 to 63 mJ/cm^2^. Here, we show the outcome of the experiments performed with a pump laser fluence of 32 mJ/cm^2^. The duration of the FEL probe pulse is ∼40 fs, whereas the laser pump pulse is about 68 fs. As shown in Ref. 27, the time jitter at FERMI is below 6 fs. The pump–probe experiment is achieved by chopping at 25 Hz the laser pump pulse. The spatial overlap between FEL and laser is done using a YAG screen, viewed with a tele-objective from a window at 40° from the FEL entrance. The temporal overlap is performed preliminarily with an antenna, and later accurately by performing a FEL-pump laser-probe experiment on GaAs. In Fig. 4(a), we show the pump-induced changes of the Kerr rotation angle at positive, $\Delta \theta_{K}^{+}(t)$, and negative, $\Delta \theta_{K}^{-}(t)$, applied magnetic fields (±50 mT). The reflectivity changes are shown in Fig. 4(b). A time-trace can be collected in less than 10 min. As standard, in Fig. 4(c), we reconstruct the relative magnetization dynamics ΔM/M simply by

$$\frac{\Delta M}{M}=100\times \frac{\Delta \theta_{K}^{+}(t)-\Delta \theta_{K}^{-}(t)}{\theta_{K,\mathrm{sat}}^{+}-\theta_{K,\mathrm{sat}}^{-}},$$ (2)

where $\theta_{K,\mathrm{sat}}^{+}$ and $\theta_{K,\mathrm{sat}}^{-}$ are the average Kerr rotation at saturation, respectively, at positive and negative fields [see the arrows in Fig. 3(a)]. We fitted the demagnetization curve with the following exponential function:

$$f(t)\propto (1-e^{-t/\tau_{\mathrm{demag}}})\;e^{-t/\tau_{\mathrm{decay}}},$$ (3)

where *τ_demag_* and *τ_decay_* are the demagnetization time and the recovery time, respectively. From the fit, *τ_demag_* is 150 ± 50 fs, hence close to the 180 fs reported in Ref. 28 and the 220 fs reported in Ref. 29. We limit our comparison to the demagnetization time, since the recovery time strongly depends on the laser pump fluence and on the Ni_*x*_Fe_1−*x*_ Permalloy stoichiometry.^28^ A detailed investigation of the demagnetization curve is already subject of existing literature,^29^ and it goes beyond the scope of present work.

## VIII. DISCUSSION

The main advantages of the TONIX polarimeter are (1) no mechanical displacement is needed after the preliminary alignment as a means of measure the FEL polarization angle, and (2) the high-efficient low-noise detection allows for polarization estimates with one pulse only. Accordingly, the experiments can be carried in shorter time.

## IX. CONCLUSION

The TONIX is an energy tunable polarimeter designed specifically for ultrashort EUV pulses and pump–probe experiments. Its high detection efficiency allows for the single-pulse determination of the x-ray polarization. We had shown the performances of the TONIX by time-resolved Kerr measurements on a Ni_80_Fe_20_ Permalloy film. We envisage the use of the TONIX also for probing Faraday rotation or natural birefringence,^18^ since both effects are large in the EUV range.

## Data Availability

The data that support the findings of this study are available from the corresponding author upon reasonable request.
